# Mucormycosis in immunochallenged patients

**DOI:** 10.4103/0974-2700.42203

**Published:** 2008

**Authors:** Jane Pak, Veronica T Tucci, Albert L Vincent, Ramon L Sandin, John N Greene

**Affiliations:** University of South Florida College of Medicine, 12901 Bruce B. Downs Blvd., Tampa, Florida 33612; 1 Division of Infectious Diseases and International Medicine, Department of Internal Medicine, College of Medicine, University of South Florida, 12901 Bruce B. Downs Blvd., Tampa, Florida 33612–4742, USA; 2 Department of Medicine, Division of Infectious Tropical Diseases, H. Lee Moffitt Cancer Center and Research Institute; Associate Professor of Pathology and Interdisciplinary Oncology, University of South Florida College of Medicine, 12902 Magnolia Drive, Tampa, Florida 33612–9497, USA

**Keywords:** Fungal infections, Mucorales, mucormycosis

## Abstract

Mucorales species are deadly opportunistic fungi with a rapidly invasive nature. A rare disease, mucormycosis is most commonly reported in patients with diabetes mellitus, because the favorable carbohydrate-rich environment allows the Mucorales fungi to flourish, especially in the setting of ketoacidosis. However, case reports over the past 20 years show that a growing number of cases of mucormycosis are occurring during treatment following bone marrow transplants (BMT) and hematological malignancies (HM) such as leukemia and lymphoma. This is due to the prolonged treatment of these patients with steroids and immunosuppressive agents. Liposomal amphotericin B treatment and posaconazole are two pharmacologic agents that seem to be effective against mucormycosis, but the inherently rapid onset and course of the disease, in conjunction with the difficulty in correctly identifying it, hinder prompt institution of appropriate antifungal therapy. This review of the literature discusses the clinical presentation, diagnosis, and treatment of mucormycosis among the BMT and HM populations.

Mucormycosis is a rare and rapidly progressive opportunistic fungal infection that most often presents among patients with diabetes mellitus, immunodeficiency, neutropenia, iron overload, and severe burns. There has been a reported rise in mucormycosis among patients with hematological malignancies (HM), e.g., acute lymphoblastic leukemia (ALL), acute myeloid leukemia (AML) and chronic myeloid leukemia (CML), and in bone marrow transplant (BMT) recipients due to the neutropenia and immunodeficiency associated with chemotherapy and post-transplant steroid treatment.[1–3] Due to the difficulty in early diagnosis and the especially detrimental course in leukemia and BMT patients, there is an urgent need to develop new methods for the rapid diagnosis and treatment of this disease.

Though a relatively uncommon fungal infection in comparison with candidiasis and aspergillosis, mucormycosis cases have been on the rise over the past decade.[4] One study indicates that the increase is due to the the increased use of chemotherapy and steroids, which is associated with a prolonged immunocompromised state.[5] Mucormycosis can be caused by any fungal species within the Mucorales order. This mold infection is also referred to as zygomycosis or phycomycosis in the literature. The most commonly seen genera in mucormycosis cases are *Rhizopus*, *Rhizomucor*, *Mucor*, and *Absidia*.[6,7] Mucormycosis is unique among the filamentous fungi because of its capacity to cause disease in a much wider human population than the other opportunistic fungi.

## ETIOLOGY

**Figure 1:**
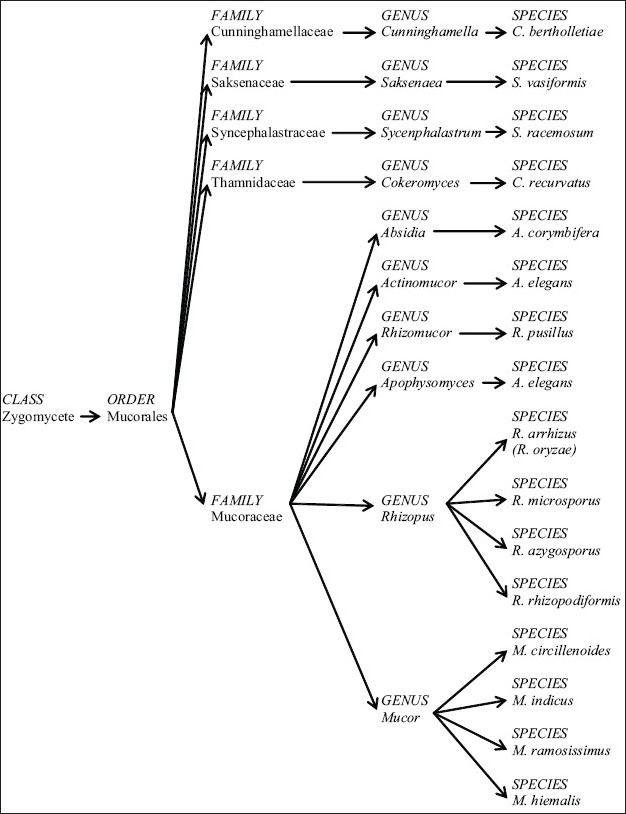
Taxonomy of the most clinically relevant Mucorales species categorized by family and genus.[75]

Mucorales, first described in 1885 by Paltauf,[8] is the largest and most widely studied order of the fungal class Zygomycete. The most commonly seen genera in mucormycosis are from the Mucoraceae family; recently, however, an increasing number of *Cunninghamella* infections from the Cunninghamellaceae family are also being reported. Figure 1 shows the classification of the clinically relevant families, genera, and species of the Mucorales order.

Mucorales fungi are characterized by fast-growing fibrous mycelium and thin-walled aseptate or hyposeptate hyphae that measure 10–20 µm in width. Right-angled branching is also seen as it rapidly grows within the host tissue. As the hyphae grow they invade the blood vessels and result in tissue infarction.[7,9] This ultimately leads to tissue necrosis and vessel thrombosis.[7,10]

Although it is an uncommon infection in the general population, exposure to Mucorales is common. Most species are saprotrophic, obtaining their nutrients from dead organic matter, and grow in soil, fruit, bread, and plants. Some are characterized as parasites of animals, plants, and other fungi. Mucormycosis infection in humans is usually acquired through airborne fungal spores.[7,10] The number of spores rapidly increases in carbohydrate-rich environments. Increased availability of soluble iron in the host is also believed to play a role in promoting Mucorales growth, especially in patients receiving deferoxamine iron chelation therapy, where the deferoxamine acts as a siderophore so the fungi may utilize the iron in its growth.[11–13]

For optimal growth in humans, Mucorales fungi require the host to have decreased levels of neutrophils. Neutrophils are the key defense against the fungi in the host tissue. In healthy humans with intact immune systems, neutrophils phagocytize these pathogens. However, in neutropenic individuals, the unregulated pathogens proliferate in the host tissue and infection ensues. Neutropenia is often a side effect of chemotherapy in cancer patients or of the malignancy itself and is also seen in immunocompromised individuals. The vast majority of patients with mucormycosis are neutropenic, with neutrophil counts usually below 0.5 × 10^9^/l. The reference range is 1.8 × 10^9^/l to 7 × 10^9^/l.

## EPIDEMIOLOGY

The literature reports an increase in mucormycosis, as compared to invasive aspergillosis, among immunocompromised patients over the last 2 decades.[14] Marr *et al.*, based on their study of 5589 transplant patients at the Fred Hutchinson Cancer Center, report that the number of cases of mucormycosis have more than doubled from 1985–1989 to 1995–1999.[1] *Rhizopus* are the most commonly isolated species among these patients. Less common, but increasingly frequent in the general population, is the *Cunninghamella bertholletiae* species.[15,16] No studies have indicated any bias for or against any specific gender, race, or age.

According to one study, the annual incidence of mucormycosis in the general population is 1.7 cases for every 1 million individuals, which amounts to approximately 500 affected Americans each year.[17] However, this is likely to be an underestimate because of the low rate (23–50%) of antemortem diagnosis[18] and also because a large number of cases remain undiagnosed due to the decline in autopsies in the United States and Europe from around 60% in the 1960s to around 10% at present.[19–21] Although most cases of mucormycosis are seen in patients with unregulated diabetes mellitus, the numbers have been decreasing over the past 20 years and, instead, an increasing numbers of individuals with HM are presenting with the infection. Recent studies indicate that 1–3% of BMT patients present with mucormycosis.[10,22,23] The incidence of mucormycosis in adult leukemia patients is reported at around 2%.[24] Those leukemia patients who have developed neutropenia due to chemotherapy or the malignancy are most susceptible to the fungus. Mortality rates in patients with HM who have mucormycosis is greater than 50%.[3,25]

Table 1 details several case reports culled from PubMed on June 16, 2007, reporting individual occurrences of mucormycosis in BMT and HM patients. Of the 34 cases presented, 10 patients (29.4%) survived the infection with successful treatment, 19 patients (55.9%) died due to ineffective treatment of the infection, and the remaining 5 (14.7%) died due to other infections or disease processes (though autopsy showed that the Mucorales infection had been successfully eradicated). These high mortality figures are similar to those reported by Kara *et al.* in a 5-year (2001–2005) retrospective study of 20 HM patients admitted to the Medical Oncology Division at the Cukurova University Hospital in Turkey[25]; the authors reported a mortality rate of 55% and a successful treatment rate of 40% in their series.[25]

**Table 1:** Individually reported mucormycosis cases in hematological malignancy and bone marrow transplant patients

Year	Author	Predisposing factor	Clinical presentation	Mucorales general/species	Positive diagnostic methods	Survive(S)/Expire(E)/Other(O)
2006	Salonen[26]	ALL	Disseminated–*lungs*,*brain*	Unspecified	Histological, biopsy	O
2006	Saito *et al*.[27]	AML	Disseminated–*lungs*, *heart*, *liver*, *spleen*, *kidneys*, *adrenals*, *and gut*	Unspecified	Post-morten histological, biopsy	E
2005	Bethge *et al*.[28]	AML	Cutaneous	*Absidia*	Biopsy, culture	S
		AML	Rhinocerebral	*Rhizopus*	Biopsy, PCR	E
		MM	Disseminated–*lungs*, *heart*, *spine*, *brain*	*Rhizopus*	Post-mortem PCR	E
		MM	Disseminated–*calf*, *lungs*	*Rhizomucor*	Post-mortem culture	E
		CLL	Pulmonary	*Rhizomucor*	Post-mortem culture	E
		ALL	Disseminated–*lungs*, *bowel*, *uterus*, *bladder*	*Rhizopus*	Histology, culture	E
2005	Barron *et al*.[29]	Hodgkin's lymphoma	Rhinocerebral	*Rhizopus*	Nasal wash culture, histology	S
2002	Cloughley *et al*.[30]	Aplastic anemia	Cutaneous	*A corymbifera*	Histological, culture	S
2002	Lee *et al*.[31]	AML, BMT	Disseminated–*lungs*, *spleen*, *kidney*	Unspecified	Histological	S
		CML, BMT	Disseminated–*spleen*, *brain*	Unspecified	Histological	E
2001	Maddox *et al*.[32]	AML, BMT	Pulmonary	*Rhizopus*	Histological, culture	E
2001	Hadithi *et al*.[33]	AML	Cutaneous	*A corymbifera*	Histological, culture	S
2000	Suh *et al*.[34]	ALL	Gastrointestinal	Unspecified	Histological	E
2000	Paterson *et al*.[35]	CML, BMT	CNS	*A corymbifera*	Microbiology, post-morterm culture	E
1999	Maertens *et al*.[23]	All, BMT	Pulmonary	*Rhizopus*	Post-mortem histological, culture	E
		CML, BMT	Pulmonary	*R microsporus*	Post-mortem histological, culture	E
		AML, BMT	Pulmonary	*Rhizopus*	Bronchoscopy culture	E
		AML, BMT	Cutaneous	*R rhizopodiformis*	Culture, histological	O
		AML, BMT	Gastrointestinal	Unspecified	Endoscopic culture, histological	S
1999	Leleu *et al*.[36]	CML, BMT	Pulmonary	*A corymbifera*	Histological, culture	O
1999	Birchall *et al*.[37]	ALL	CNS	Unspecified	Histological	S
1998	Peñalver *et al*.[38]	CML, BMT	Rhinocerebral	*Rhizopus*	Histological, biopsy	S
1996	Jantunen *et al*.[39]	BMT	Cutaneous	*A corymbifera*	Culture, biopsy	S
1997	Leong *et al*.[40]	Aplastic anemia	Cutaneous	*A corymbifera*	Histological	S
1996	Funada and Matsuda[24]	CML	Pulmonary	Unspecified	Post-mortem culture	E
		AML	Pulmonary	Unspecified	Transbronchial lung biopsy	O
		AML	Pulmonary	Unspecified	Postmortem culture	O
		AML	Pulmonary	Unspecified	Transbronchial lung biopsy	O
		AML	Pulmonary	Unspecified	Postmortem culture	E
		CML	Pulmonary	Unspecified	Postmortem culture	E
		CML	Pulmonary	Unspecified	Postmortem culture	E
1985	Benbow *et al*.[41]	AML	Disseminated–*face*, *brain*, *liver*, *spleen*, *kidneys*, *esophagus*	*R arrhizus*	Postmortem histological, culture	E

ALL: Acute Lymphoblastic leukemia; AML: Acute Myeloid Leukemia; BMT: Bone Marrow Transplant; CML: Chronic Myeloid Leukemia; CMV: Cytomegalovirus; CNS: Central Nervous System; MM: Multiple Myeloma; O: Death caused by Other Complicating disease processes with no Mucorales on autopsy; PCR: Polymerase Chain Reaction

## CLINICAL PRESENTATION

Mucormycosis commonly presents in five forms: it may affect the pulmonary, rhinocerebral, gastrointestinal, cutaneous, or the central nervous systems.[7] Dissemination may also occur due to the highly invasive nature of Mucorales.[7] Dissemination is a term used to describe an infection that affects two or more noncontiguous organ systems. Of the five forms, pulmonary and rhinocerebral are the most common. Disseminated mucormycosis affects the lungs and brain most often, with the infection usually originating in the lungs.[6] Our review of the case reports in the literature suggest that among HM and BMT patients the pulmonary presentation of mucormycosis is most common, followed by the disseminated form. A study by Lee *et al.* of 87 pulmonary mucormycosis patients indicated that those with HM comprised 32% of the cases; the majority (56%) of the cases were those with diabetes mellitus.[5] Table 2 details the different features associated with each of the five forms of the infection and the common differential diagnoses for each.

**Table 2:** Clinical information divided by the most common clinical presentations

	Signs and symptoms	Differential diagnoses	Prevalence among BMT and HM patients	Other comments
**Pulmonary mucormycosis**	Pneumonia, fevers, rales, decreased breath sounds, hemoptysis, difficulty breathing, infiltrates in lungs	Aspergillosis, pulmonary embolism	Most common among leukemia patients	-
**Rhinocerebral mucormycosis**	Orbital swelling, cellulitis, black nasal discharge, loss of vision, ptosis, proptosis, headache, necrotic ulceration, fever	Bacterial orbital cellulitis, bacterial cavernous sinus thromboisis, aspergillosis, rapidly growing orbital tumor	Not as common	Presents mostly in diabetic patients[5]
**Gastronintestinal mucormycosis**	Tenderness to abdominal palpation, abdomianl mass, peritonitis (associated with ruptures)	Bowel obstruction, ileocecal tuberculosis	Not as common	Presents mostly among malnourished adults and with other underlying gastrontestinal diseases
**Cutaneous mucormycosis**	Black necrotic lesions	Anthrax, ecthyma gangrenosa associated with pseudomonal infection	Common	Favorable outcomes with surgery and skin grafts
**Central nervous system mucormycosis**	Decreased conscikousness, ear pain, convulsions, paralysis, cranial nerve deficits	Cranial nerve palsies, stroke, otitis extema	Not as common	-

BMT: Bone Marrow Transplant; HM; Hematological Malignancy

The most common symptom in pulmonary mucormycosis is a high-grade fever (> 39°C) that does not subside despite the administration of antibiotics.[5,16,24,26,32,36,42–47] Coughing and respiratory distress are also seen. Auscultation of these patients' chests may reveal moist rales and pleural friction rubs. Pleuritic chest pain is often present in these patients. Oral thrush may also appear. Computed tomography of the chest may show ill-defined round densities, halo and lobar expansion, and pleural infiltrates. Patchy consolidations and cavitations may also be seen on chest radiography.

Cases of rhinocerebral mucormycosis present with facial pain, periorbital cellulitis, proptosis, visual deficiencies, black necrotic lesions and discharge from the nasal and palatal mucosa, and fever.[38,48,49] Biopsy reveals fungal hyposeptated hyphae consistent with Mucorales. Most patients with this form of mucormycosis have diabetes mellitus[5]; it is not very common among BMT and HM patients.

Gastrointestinal mucormycosis presents with features similar to that seen in bowel obstruction. Tenderness on palpation, with or without the presence of a mass, and hepatomegaly or splenomegaly have been reported.[23,34] This form of the infection is seen most commonly among malnourished adults and in individuals with underlying gastrointestinal disease or abnormalities such as kwashiorkor, colitis, typhoid, or pellagra.[2]

Cutaneous involvement by mucormycosis has been reported as presenting with black necrotic lesions, most often on the face and extremities. Many of the cases of cutaneous mucormycosis reported in BMT and HM patients seem to have been caused by *A corymbifera*. The site of the lesion may be where catheters are placed.[40] Reports in the literature suggest that due to the superficial nature of these infections, early diagnosis and surgical debridement with skin grafting give highly favorable outcomes.[23,30,33,40]

Brain mucormycosis is not common by itself; CNS involvement is usually a result of dissemination of the fungal infection from pulmonary or rhinocerebral sources.[37] Paterson *et al.* have reported a CML patient in whom mucormycosis spread through the ear to invade cerebral tissue; the patient died 15 days post-BMT.[35]

## DIAGNOSIS

Mucormycosis can be a fatal infection and early diagnosis and treatment are of extreme importance for successful eradication of the infection and for patient survival. Differential diagnoses may be varied due to the different clinical presentations and organ systems involved. The most commonly employed technique for differentiating between mucormycosis and other diseases is a histopathological study.[50] However, due to the frequently encountered difficulty in distinguishing Mucorales from other filamentous fungi,[9] more sophisticated diagnostic techniques are emerging and being evaluated. A discussion of the various techniques available follows. Table 3 summarizes their general features, issues, and efficacy.

**Table 3:** Diagnostic technique for mucormycosis

Diagnostic technique	General features	Issues	Limited evaluation	Efficacy
Histopathology	Most commonly used; Grocott-Gomori methednamine silver (GMS) or periodic acid-Schiff (PAS) stain most effective; Bronchoalveolar lavage may be especially Useful in pulmonary cases	Mucorales may be indistinguishable from other filamentous fungi unles experienced laboratories to the examination	-	Moderately high
Culture	Not used alone in diagnosis due to the fact that saprophytic mold does not always indicate the occurrence of an infection; Bronchoalveolar lavage may be especially Useful in pulmonary cases	Frequency produces false negatives unless tissues are minced, not ground	-	Low
MicroSeq D_2_ Ribosomal DNA sequencing	Used in other filamentous fungi with a good deal of success	More work remains to make it an effective diagnostic technic for mucormycosis	x	Low
PCR	Two nesterd PCR assays especially useful for differentiating between aspergillosis and mucormycosis	Not widely used	x	High
ITS_2_ sequencing	Uses variability between fungi in their internal transcribed spacer 2 sequences for diagnosis	Not widely used	x	High
Serological testing	Tests for mucor IgE antibodies	More work remains to make it an effective diagnostic technique for mucormycosis	x	Unknown
Susceptibility testing-Fluorescenece-based microplate assays	Used in *Aspergillus* with good deal of success	More work remains to make it an effective diagnostic technique for mucormycosis	x	Unknown
Susceptibility testing-XTT assays	Produces results similar to older susceptibility testing methods but in less tiome and on multiple drugs; more viable for use in most laboratories	Not widely used	x	Moderately high

PCR: Polymerase Chain Reaction; XTT: tetrazolium salt 2,3-bis {2-methoxy-4-nitro-5-[(sulfenylamino)carbonyl]-2H-tetrazolium-hydroxide}

For a definitive diagnosis of mucormycosis, the histopathological specimen need not be of the isolated fungus, but may be a sample of the necrotic tissue from the site in question. The Grocott-Gomori methenamine silver (GMS) stain is most effective for identifying fungi. However, hematoxylin and eosin, periodic acid-Schiff (PAS), or Calcofluor White stains may also be used.[2] Invasion of the tissue by fungal hyphae and right-angle branching will be present in mucormycosis specimens.[9] Fungal angioinvasion, perineural invasion, and the presence of neutrophilic infiltrate in the necrotic tissue are also characteristic of mucormycosis.[9] Mucorales are sometimes difficult to distinguish in tissues from other filamentous fungi on histopathological examination.[9] Nevertheless, histopathology continues to be commonly employed for the diagnosis of mucormycosis.

Culture studies often fail to grow Mucorales in mucormycosis patients. Blood cultures are especially known to produce false negative results. The ideal culturing technique is to receive the infarcted tissue and submit it for processing by mincing the tissue with a sterile scalpel and not by grinding it. The syncytial cells of the Mucorales fail to grow in culture if submitted to a grinding procedure, which will produce small fragments devoid of any enclosing septal cell walls. Mucorales grow well on Sabouraud dextrose, brain-heart infusion, or potato dextrose agar incubated at 25–30°C. Identification of saprophytic mold does not conclusively indicate that an infection with that organism has occurred; therefore culture is not usually used alone for diagnosis.

A recent Mayo clinic study using the MicroSeq D2 large-subunit ribosomal DNA sequencing kit to identify filamentous fungi concluded that nucleic acid sequencing using this technique holds much promise, with accurate identification possible in under 24 h.[51] However, more work needs to be done to improve its relevance to the more clinically prevalent fungal species. Less than 50% of the Zygomycetes tested in this study were identified correctly. Kobayashi *et al.* recently conducted a study showing the usefulness of panfungal PCR on serial serum samples for diagnosing pulmonary mucormycosis caused by *Cunninghamella bertholletiae*.[45] Rickerts *et al.* reported the use of a combination of two nested PCR assays to differentiate between *Aspergillus* and Zygomycetes (which are difficult to tell apart by clinical and radiographic signs) and successfully identified the Zygomycetes to the species or genus level.[52] They were able to identify the organism in culture-negative samples as well. Another technique that has been recently developed takes advantage of the sequence variability in the internal transcribed spacer 2 (ITS2) of fungi for identifying Zygomycete isolates to the species level; this is possible because of the significantly different sequences between species.[53,54]

At present, serological testing is not recommended for diagnosis due to its investigational status.[55] Only a few reported cases have used antibody testing for mucor IgE in order to diagnose mucormycosis.[56]

Susceptibility testing is used to determine a drug's potential effectiveness for eradicating infection and inhibiting growth of the pathogen. One susceptibility testing method commonly used for *Aspergillus* utilizes fluorescence-based microplate assays. This method may come into use for Zygomycetes in the future but has not yet been comprehensively evaluated. A recent study by Antachopoulos showed that clinically important Zygomycetes show significant metabolic activity in XTT assays even before visual or spectrophotometric evidence of activity becomes apparent.[57] XTT is the tetrazolium salt 2,3-bis{2-methoxy-4-nitro-5-[(sulfenylamino) carbonyl]-2H-tetrazolium-hydroxide}. The XTT assay is a colorimetric method of quantifying fungal growth by measuring the metabolism of XTT by fungal mitochondrial dehydrogenases. Electron transfer agents such as menadione are also needed for efficient reduction.[57] These XTT assays, performed at 6-12 h post-inoculation, provided results in agreement with the older standard methods of susceptibility testing, which are done 24 h post-inoculation. Several other methods exist for rapid susceptibility testing in filamentous fungi, however many are purely for assessing susceptibility to amphotericin B and require the use of expensive laboratory equipment or potentially hazardous substances. In contrast, XTT assays may also be used for testing susceptibility to other drugs and is a more viable alternative for use in most laboratories.

## TREATMENT

Mortality rates among patients with HM who have mucormycosis remain high despite antifungal therapy. Surgical intervention and debridement is the gold standard of treatment for eradicating the infection.[4,7,58] However many of the clinical presentations do not allow for such methods of treatment. Favored alternatives for treatment include various amphotericin B formulations and posaconazole, with different Mucorales genera and species showing slight differences in susceptibilities to the drugs.[59] Most cases that have been successfully treated have required approximately 5 weeks of therapy.

There are many similarities in the clinical presentations of aspergillosis and mucormycosis. The ineffectiveness of the drugs used in aspergillosis for the treatment of mucormycosis makes it especially important to establish the diagnoses as early as possible in the course of disease. For leukemia and BMT patients who receive prophylactic antifungal drugs as part of their treatment, it is crucial to distinguish between the different fungal infections as soon as possible after symptoms appear in order to administer the correct antifungal therapy. Early correction of predisposing factors such as neutropenia and immunodeficiency are also important in controlling the progression of the infection.

The most effective treatments for mucormycosis in BMT and leukemia patients are various formulations of amphotericin B combined with posaconazole.[59] Itraconazole is somewhat ineffective against Mucorales, especially against *Rhizopus* species.[6,59] there is conflicting data on the efficacy of echinocandins against mucormycosis and the reported cases do not present a strong argument either way.[60,61]

Voriconazole, an effective drug in aspergillosis,[62] is not recommended against mucormycosis due to its ineffectiveness both *in vitro* and *in vivo*.[22,46,59,63–65] Not only is voriconazole ineffective in the treatment of mucormycosis, it has also been associated with breakthrough infections of mucormycosis.[22,63,64,66] Several cases have been published where patients with HM were given voriconazole against *Aspergillus* infection, only to find that they develop mucormycosis later. These cases showed breakthrough mucormycosis at 9–12 days after voriconazole administration was started as an empiric antifungal treatment in leukemia. Marty *et al.* also reports breakthrough mucormycosis in stem cell transplant recipients who received voriconazole as prophylactic or empiric therapy.[22] Voriconazole is an effective drug against *Aspergillus*, *Candida*, *Scedosporium*, and *Fusarium*.[67,68] The popular utilization of this drug for prophylaxis against common fungal agents allows the Mucorales (which are resistant to it) to emerge unchallenged and infect these highly immunocompromised patients.[66] These findings emphasize the need for more caution when using voriconazole as a prophylactic agent in this patient population.

Amphotericin B has been the drug of choice against mucormycosis for over 50 years due to its superior effectiveness compared to other therapies. Treatment duration is usually between 3 and 6 weeks and the total dose that needs to be administered ranges from 2.0–4.0 g, depending on the specific case.[7] Among the different formulations of this drug are liposomal amphotericin B (LAmB) and cochleate-containing amphotericin B (CAmB). LAmB is relatively low in toxicity and does not have many adverse effects, making it the favored choice. Salonen described one leukemia patient in whom LAmB therapy was concurrently administered with chemotherapy.[26] An allogeneic bone marrow transplantation had been done in this patient and it was possible to prevent relapses of mucormycosis throughout the treatment period by continuing LAmB in conjunction with secondary antifungal prophylaxis. According to Wingard *et al.*, administration of CAmB to treat mucormycosis may not be favorable and may even be toxic due to the high dosage levels and prolonged treatment required.[69] Another lipid formulation of amphotericin B, amphotericin B lipid complex (ABLC), has had success in treating patients with mucormycosis.

Posaconazole is a relatively new drug that appears to be a promising alternative or adjunctive to LAmB administration.[59,70,71] Rickerts *et al.* reported the case of a leukemia patient in whom disseminated mucormycosis was successfully treated with the use of a combination of LAmB and posaconazole without surgical intervention. Other authors have also reported similar success.[62,72] Many experienced conditions favor combination therapy of posaconazole and lipid formulations of amphotericin B over monotherapy. However, there is no conclusive data yet available to determine if combination therapy is additive, synergistic, or antagonistic.

Another alternative or adjuvant therapy is hyperbaric oxygen (HBO). A study published in 2004 by Garcia-Covarrubias showed 60% survival rate using HBO as an adjuvant therapy to amphotericin B and surgical debridement.[73] A 1994 study of 208 cases by Yohai also supported the improvement in prognosis with the use of HBO.[74] Despite its promising outlook, the literature is still too sparse in this area to make a conclusive case for including HBO as part of the treatment regimen.

## CONCLUSION

The high mortality rate among mucormycosis patients calls for an increased index of suspicion and aggressive attempts to diagnose and treat the disease at an early stage. Because of its rarity, mucormycosis is often not included in the differential diagnosis until widespread Mucorales invasion has already taken place in the patient. Its commonplace presence in the environment emphasizes the importance of keeping the wards and hospital instruments free of these opportunistic fungi. Increasing the implementation of promising new diagnostic methods such as PCR nested assays and ITS2 sequencing may help increase the rate of successful Mucorales identification in the early stages of infection. However, the new assays await FDA approval for implementation in hospital-based clinical microbiology laboratories. LAmB and posaconazole treatment have helped to treat many cases of mucormycosis. However, despite these advances, the incidence of the infection has risen in recent years, in part due to the use of prolonged chemotherapy and immunosuppressive treatment in HM and BMT patients. Given the increase in cases of breakthrough mucormycosis after general antifungal prophylaxis and the overall increase in cases in this population, anti-Mucorales prophylaxis studies are warranted and should be given due importance, alongside the search for better methods of diagnosis and treatment.
